# Operative volume and surgical case distribution in Uganda’s public sector: a stratified randomized evaluation of nationwide surgical capacity

**DOI:** 10.1186/s12913-019-3920-9

**Published:** 2019-02-06

**Authors:** Katherine Albutt, Maria Punchak, Peter Kayima, Didacus B. Namanya, Mark G. Shrime

**Affiliations:** 1 Program in Global Surgery and Social Change, Harvard Medical School, 641 Huntington Avenue, Boston, MA 02115 USA; 2 Department of Surgery, Massachusetts General Hospital, Boston, MA USA; 3 David Geffen School of Medicine at UCLA, Los Angeles, CA USA; 4 Mbarara University of Science and Technology, Mbarara, Uganda; 5 Ministry of Health, Kampala, Uganda; 6 Uganda Martyrs University, Nkozi, Uganda; 7 Department of Otolaryngology, Massachusetts Eye and Ear Infirmary, Boston, MA USA

**Keywords:** Global surgery, Operative volume, Case distribution, Uganda, Surgical workforce

## Abstract

**Background:**

Little is known about operative volume, distribution of cases, or capacity of the public sector to deliver essential surgical services in Uganda.

**Methods:**

A standardized mixed-methods surgical assessment and retrospective operative logbook review were completed at 16 randomly selected public hospitals serving 64·0% of Uganda’s population.

**Results:**

A total of 3014 operations were recorded, annualizing to a surgical volume of 36,670 cases/year or 144·5 operations/100,000people/year. Absolute surgical volume was greater at regional referral than general hospitals (*p* < 0·001); but, relative surgical volume/catchment population was greater at the general versus regional level (*p* = 0·03). Most patients undergoing operations were women (78·3%) with a mean age of 26·9 years. The overall case distribution was 69·0% obstetrics/gynecology, 23·7% general surgery, 4·0% orthopedics, and 3·3% other subspecialties. Cesarean sections were the most common operation (55·8%). Monthly operative volume was strongly predicted by number of surgical, anesthetic, and obstetric physician providers (훽=10·72, *p* = 0·005, R^2^ = 0·94) when controlling for confounders. Notably, operative volume was not correlated with availability of electricity, oxygen, light source, suction, blood, instruments, suture, gloves, intravenous fluid, or antibiotics.

**Conclusion:**

An understanding of operative case volume and distribution is essential in facilitating targeted interventions to strengthen surgical capacity. These data suggest that surgical workforce is the critical driver of operative volume in the Ugandan public sector. Investment in the surgical workforce is imperative to ensure access to safe, timely, and affordable surgical and anaesthesia care.

## Background

Surgery is an integral, indivisible component of healthcare, yet an estimated 5 billion people worldwide currently lack access to safe, timely, and affordable surgical, obstetric, and anaesthesia care [1, 2]. The provision of surgical, anaesthetic, and obstetric care is essential in achieving universal health coverage, Sustainable Development Goal Target 3.8. In 2015, the *Lancet* Commission on Global Surgery (LCoGS) was convened to assemble evidence of the state of surgical care worldwide and examine potential solutions to narrow the surgical access chasm [1]. The Commission recommended all countries collect six core surgical indicators which, taken together, were designed to reflect the strength and capacity of the surgical system [1]. One of the six core indicators is operative volume, defined as the number of procedures undertaken in an operating theatre per 100,000 population per year [1].

The LCoGS established that a minimum threshold of surgical volume of 5000 procedures per 100,000 population per year is associated with improved health outcomes [1, 3]. Similarly, low operative volumes are known to be associated with high mortality and morbidity from common, treatable surgical conditions [1]. Unfortunately, operative volume worldwide, and particularly in low- and middle-income countries (LMICs), falls severely below this threshold. Of the 313 million surgical procedures performed worldwide every year, only 6% were performed in countries home to the poorest 40% of the world’s population [1, 2, 4]. At minimum, an estimated 321·5 million surgical procedures per year are needed to address the current burden of surgical disease [5]. In LMICs, the best available estimates suggest that 143 million additional surgical procedures are needed each year to save lives and prevent disability [1, 5]. Minimum need has also been shown to be geographically variable, with the greatest unmet need estimated to be in eastern, western, and central sub-Saharan Africa, and south Asia [1].

Uganda, an east African country with a population of 39 million people, is plagued by low operative volume and poor access to surgical, obstetric, and anaesthesia care [6, 7]. The country’s population is served by a tiered and decentralized public healthcare system based on referral, composed of 43 primary-level hospitals - general hospitals (GHs), 14 secondary-level hospitals - regional referral hospitals (RRHs), and two tertiary-level hospitals - national referral hospitals (NRHs), one of which is exclusively for mental health [8]. While 51% of Ugandans seek healthcare from public facilities, little is known about operative volume, case distribution, and capacity of the public healthcare system to deliver essential surgical services [8].

While other nations including Madagascar, Rwanda, Sierra Leone, and Liberia have produced publications on the nationwide operative volume, literature regarding operative volume and capacity in the Ugandan public health care sector is sparse [9–12]. The only previously published study regarding operative volume in Ugandan public hospitals assessed surgical capacity at 14 hospitals and found that each hospital performed an average of 886 surgical procedures year, resulting in a rate of 154 major surgeries per 100,000 population [13]. Most providers of surgical services are concentrated in Kampala, and little is known about the capacity and operative volume elsewhere in the country. To better understand the current capacity of the healthcare system to deliver surgical care in Uganda, we sought to describe the operative volume, case mix and procedure distribution in Uganda’s public sector in order to elucidate the key drivers of operative volume.

## Methods

**Fig. 1:**
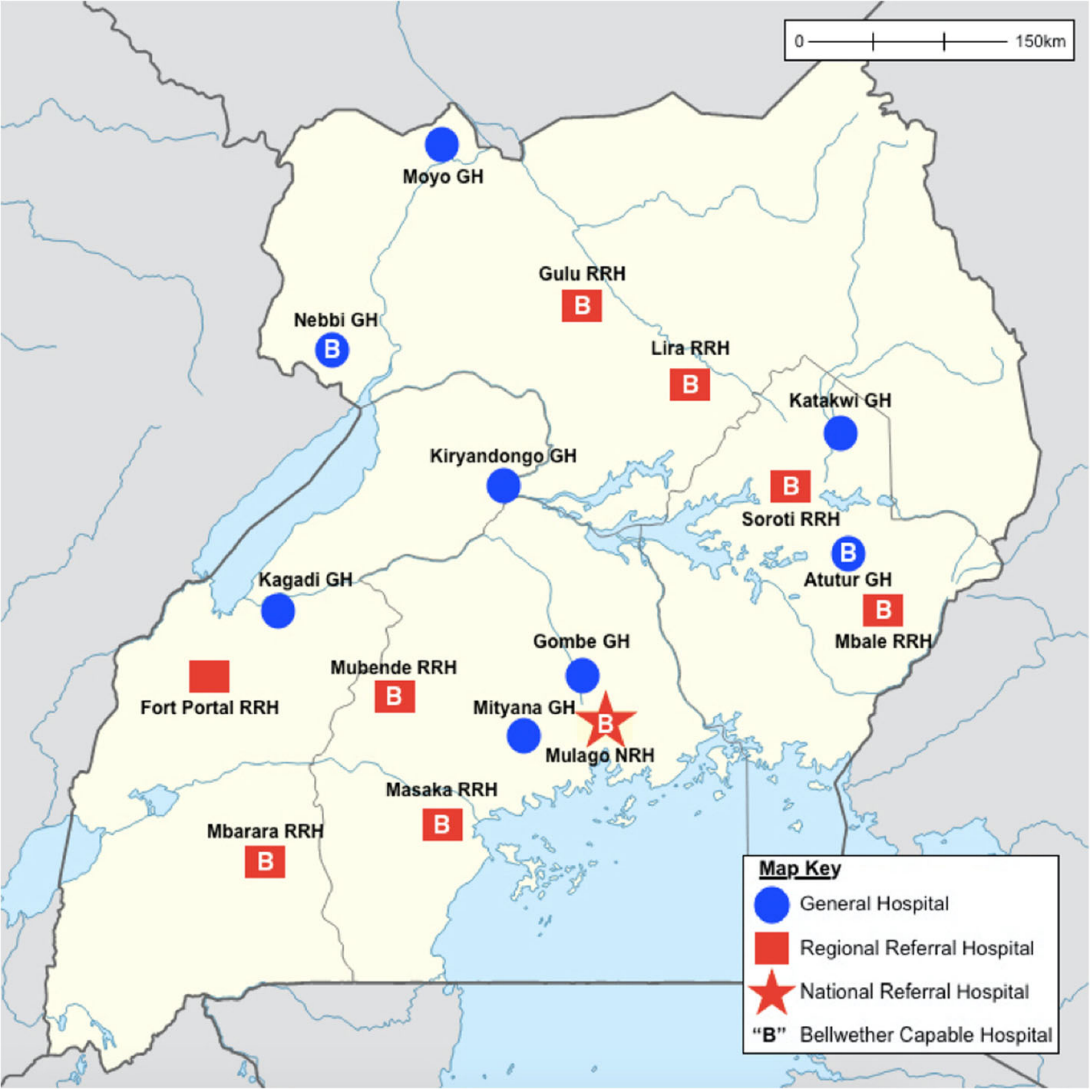
Map of surveyed facilities (source: [14])

The data presented here are derived from a mixed-methods stratified randomized evaluation of nationwide surgical capacity in Uganda’s public sector that was conducted from September to November, 2016 [14, 15]. In order to capture a representative sample of hospitals, two GHs and two RRHs within each of Uganda’s four administrative regions were randomly selected, yielding a total of 16 public hospitals for data collection. These hospitals are depicted in Fig. 1. Mulago, the national referral hospital, was also sampled during this study though excluded from this analysis due to construction rendering care at Mulago fragmented across multiple sites and inability to provide comprehensive operative log data.

### Surgical assessment tool

A mixed-methods validated Surgical Assessment Tool (SAT), jointly developed by the Harvard Program in Global Surgery and Social Change (PGSSC) and the World Health Organization (WHO), was employed at each facility [9]. The assessment consisted of hospital walk-throughs and interviews with hospital directors and providers designed to assess five domains - infrastructure, service delivery, workforce, information management, and financing. An operation was defined as “a procedure (the incision, excision, or manipulation of tissue that needs regional or general anaesthesia, or profound sedation to control pain) undertaken in an operating room” [16, 17]. A functioning operating room was defined as a room in which operations were taking place. Access to necessary inputs in the surgical system was defined as always (100% of the time), almost always (76–99% of the time), most of the time (51–75% of the time), sometimes (26–50% of the time), rarely (1–25% of the time), and never (0% of the time). Detailed quantitative and qualitative data from this mixed-methods study have been published elsewhere [15, 18]. A subsequent phase of this study evaluated surgical capacity at private and private not-for-profit hospitals, the results of which are forthcoming.

### Operative logbooks data extraction

Within Uganda, the Ministry of Health (MOH) uses the Health Management Information System (HMIS) to routinely collect information to enable planning, decision making, and monitoring and evaluation of the health care delivery system [19]. However, available evidence suggests that the Ugandan HMIS system often does not accurately capture health facility performance and thus we did not rely on the HMIS system for operative case extraction [20]. Instead, operative logbooks were retrospectively reviewed for the 30 days preceding the site visit. Logbooks have been shown to be an effective method to capture accurate operative volume in LMICs. In a recent study in Uganda, logbooks captured 99% of the operations and 94% of all deaths when comparing prospective data collection with retrospective surgical logbook review [21]. All cases conducted and recorded at selected hospitals were anonymized and coded in a comprehensive electronic database.

### Data analysis

Stata 12.1 was used for data analysis (Stata Corp, College Station, TX, USA). Fisher’s exact, Wilcoxon rank-sum and the Kruskall Wallis one-way analysis of variance test were used for comparisons. Multivariable linear regression was used to assess the relationship between operative volume and its associated drivers, accounting for confounding variables. Statistical significance was defined as *p* < 0·05. When annualizing data and extrapolating to the national level, results were scaled linearly.

### Ethical considerations

The institutional review board (IRB) at Children’s Hospital Boston deemed this study exempt. The IRB at Mbarara University of Science and Technology and the Uganda National Council for Science and Technology approved this study. Permission and verbal informed consent to participate was additionally obtained from each facility participating in the assessment. No identifying information was collected from study participants. All operative logbook data were kept in a secure location and immediately de-identified.

## Results

Based on catchment area, the 16 sampled facilities served 64.0% of the Ugandan population and accounted for 64·8% of all public hospital beds in Uganda. The total number of beds at surveyed hospitals was 4113, of which 2880 were at RRHs and 1233 at GHs. Capacity differed significantly by hospital type - GHs had an average capacity of 154.1 beds and RRHs an average capacity of 360·0 beds (*p* = 0·004). The total number of operating rooms (ORs) was 42, with 31 at RRHs and 11 at GHs. On average, there were 2·63 functioning operating rooms (ORs) per sampled facility, equating to 0·17 functioning ORs per 100,000 population. There was a significant difference between GHs, which had an average of 1·38 ORs per facility, and RRHs, which had 3·88 ORs per facility (*p* = 0.003). Nine of the 16 sampled hospitals, 7 RRHs and 2 GHs, were Bellwether facilities (defined as facilities capable of performing exploratory laparotomy, cesarean section, and open fracture repair – a proxy for surgical systems that are “functioning at a level of complexity advanced enough to do most other surgical procedures”) [1]. A total of 83 surgical, anesthetic and obstetric & gynecology physician providers (SAOs) were working at the surveyed hospitals, 72 at RRHs and 11 at GHs (*p* = 0·002). All RRHs were considered trainee hospitals, compared to zero GHs.

A total of 3014 procedures were recorded in the 30-day period prior to each site visit. Of these, 677 (22·5%) were performed at GHs and 2337 (77·5%) at RRHs. This annualizes to a surgical volume of 36,670 cases/year or 144·5 operations/100,000 people/year. Mbarara Regional Referral Hospital in western Uganda performed the greatest number of surgical procedures in the 30-day time frame. Atutur General Hospital in Eastern Uganda did not report any surgical procedures over the preceding 30 days due to ongoing surgical wing renovations (they completed an average of 11 cases per month prior to the renovation). A breakdown of operative volume by hospital can be found in Table 1.

**Table 1:** 30-Day operative volume by hospital

Hospital	Type	Beds	Functioning ORs	General Surgery	Obstetrics & Gynecology	Orthopedics	Other Subspecialties & Surgical Camps	30-Day Operative Volume
Atutur	GH	400	0	0 (N/A)	0 (N/A)	0 (N/A)	0 (N/A)	0
Gombe	GH	100	1	20 (23.5%)	65 (76.5%)	0 (0.0%)	0 (0.0%)	85
Kagadi	GH	104	1	2 (1.7%)	113 (98.3%)	1 (0.9%)	0 (0.0%)	115
Katakwi	GH	100	1	48 (57.1%)	35 (41.7%)	0 (0.0%)	0 (0.0%)	84
Kiryandongo	GH	104	3	24 (31.6%)	49 (64.5%)	3 (3.9%)	0 (0.0%)	76
Mityana	GH	150	1	20 (12.0%)	146 (88.0%)	0 (0.0%)	0 (0.0%)	166
Moyo	GH	140	1	6 (10.0%)	54 (90.0%)	0 (0.0%)	0 (0.0%)	60
Nebbi	GH	135	3	29 (31.9%)	59 (64.8%)	3 (3.3%)	0 (0.0%)	91
Fort Portal	RRH	372	5	61 (22.3%)	195 (71.4%)	3 (1.1%)	14 (5.1%)	273
Gulu	RRH	400	5	39 (23.9%)	72 (44.2%)	4 (2.5%)	48 (29.4%)	163
Lira	RRH	400	4	22 (11.2%)	168 (85.7%)	6 (3.1%)	0 (0.0%)	196
Masaka	RRH	330	5	72 (24.5)	219 (74.5%)	3 (1.0%)	0 (0.0%)	294
Mbale	RRH	454	4	91 (22.8%)	258 (64.7%)	50 (12.5%)	0 (0.0%)	399
Mbarara	RRH	451	4	186 (32.6%)	319 (56.0%)	29 (5.1%)	36 (6.3%)	570
Mubende	RRH	212	2	39 (22.2%)	134 (76.1%)	3 (1.7%)	0 (0.0%)	176
Soroti	RRH	261	2	56 (21.1%)	195 (73.3%)	15 (5.6%)	0 (0.0%)	266
Total	RRH	4113	43	715 (23.7%)	2081 (69.0%)	120 (4.0%)	98 (3.3%)	3014

Absolute surgical volume was statistically significantly greater at RRHs than GHs (*p* < 0·001), with RRHs and GHs performing 2337 and 677 surgeries in 30 days, respectively. However, relative surgical volume/catchment population was greater at the GH versus RRH level (*p* = 0·03), with 124·3 operations / 100,000 people / year at the RRH level and 328·5 at the GH level. Anaesthesia type (general anaesthesia, spinal anaesthesia, local anaesthesia, and sedation) varied significantly by hospital type (p < 0·001). Details on surgical infrastructure, operative volume, case and anaesthesia distribution by hospital level can be found in Table 2**.**

**Table 2:** Surgical infrastructure, 30-Day operative volume and case distribution by hospital level

	General Hospitals	Regional Referral Hospitals	Total	*P*-value
Number of Institutions Assessed	8	8	16	1
Total Population Served	2,507,400	22,873,000	25,380,400	< 0.001
Average Age	26.45	27.06	26.92	0.33
Gender Breakdown				< 0.001
Male	100 (14.8%)	299 (12.8%)	399 (13.2%)	
Female	564 (83.3%)	1796 (76.9%)	2360 (78.3%)	
Unknown	13 (1.9%)	242 (10.4%)	255 (8.5%)	
Average Beds / Facility	154.1	360.0	257.1	0.004
Average ORs / Facility	1.38	3.88	2.63	0.003
Bellwether Facilities	2 (22.2%^a^)	7 (77.8%^a^)	9 (100.0%^a^)	0.04
Total SAOs	11 (13.3%^a^)	72 (86.8%^a^)	83 (100.0%^a^)	0.002
Trainee Hospitals	0 (0.0%)	8 (100.0%^a^)	8 (100.0%^a^)	< 0.001
Average Cases/100,000 Population/Year	328.5	124.3	144.5	0.03
Case Breakdown (30 Day Operative Log Total)	677 (22.5%^a^)	2337 (77.5%^a^)	3014 (100.0%^a^)	< 0.001
General Surgery	149 (22.0%)	566 (24.2%)	715 (23.7%)	
Ob/Gyn	521 (77.0%)	1560 (66.8%)	2081 (69.0%)	
Ortho	7 (1.0%)	113 (4.8%)	120 (4.0%)	
Neuro	0 (0.0%)	12 (0.5%)	12 (0.4%)	
Surgical Camp	0 (0.0%)	86 (3.7%)	86 (2.9%)	
Anaesthesia Breakdown				< 0.001
General Anaesthesia	185 (27.3%)	716 (30.6%)	901 (29.9%)	
Spinal Anaesthesia	300 (44.3%)	1425 (61.0%)	1725 (57.2%)	
Local Anaesthesia	88 (13.0%)	111 (4.8%)	199 (6.6%)	
Sedation	52 (7.7%)	22 (0.9%)	74 (2.4%)	
Unknown	52 (7.7%)	63 (2.7%)	115 (3.8%)	

% reported as a % of total operations within that level of facility except where indicated by ^a^ where reported as % of overall total

**Fig. 2:**
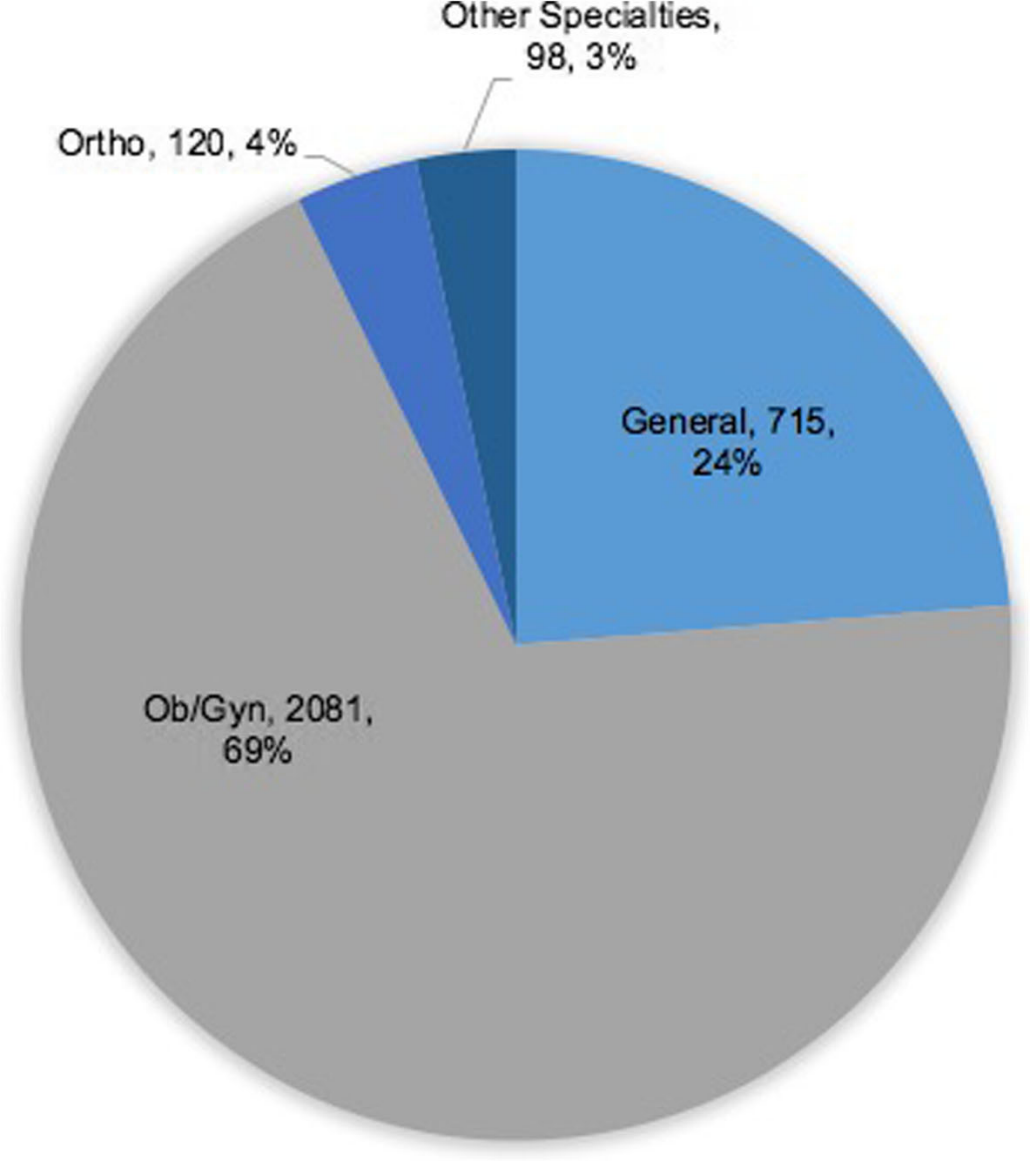
Surgical case type distribution at surveyed facilities

**Fig. 3:**
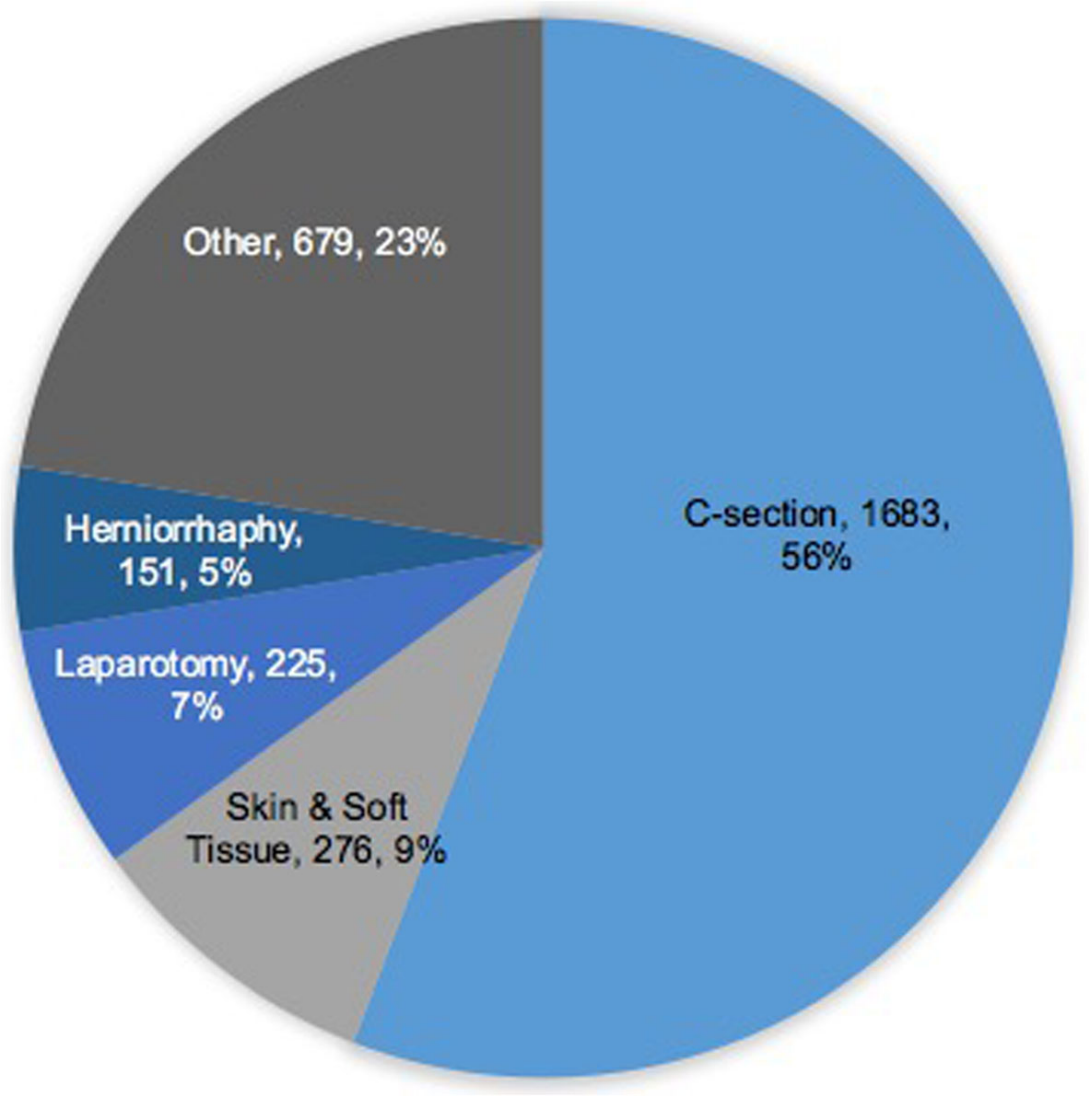
Surgical procedure distribution at surveyed facilities

Overall case distribution was 69·0% obstetrics/gynecology, 23·7% general surgery, 4·0% orthopedics, and 3·3% other subspecialties, and varied significantly by hospital type [15]. Details of case distribution by specialty can be seen in Fig. 2. Most patients undergoing operations were women (78·3%) with a mean age of 26·9 years with no statistical difference between GHs and RRHs. Cesarean sections were the most common operation (55·8%), followed by skin/soft tissue (9·2%), laparotomy (7·5%), and herniorraphy (5·0%). Case volume breakdown by operation can be seen in Fig. 3.

**Fig. 4:**
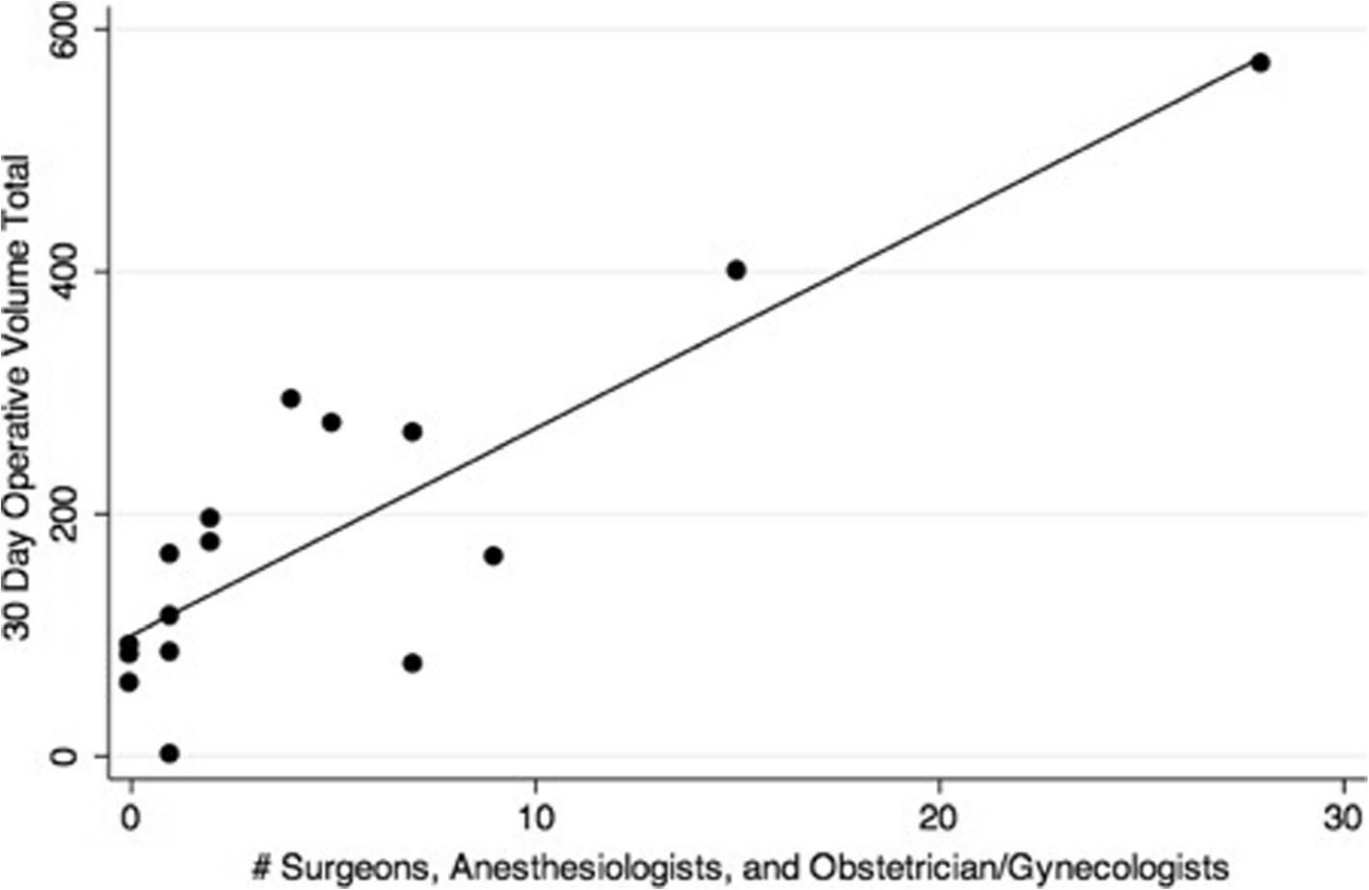
Correlation between number of surgery, anaesthesia and ob/gyn providers and operative volume among the surveyed facilities, controlling for confounders (model was adjusted for # hospitals beds, # ORs, presence of trainees, availability of infrastructure, medications, blood and OR equipment)

When univariate analysis was performed, operative volume was strongly predicted by number of SAO providers (*β*=17·1, *p* < 0·001), number of hospital beds (*β*=0·7, *p* = 0·008), number of ORs (*β*=52·4, p = 0·01), hospital level (*β*=207·5, p = 0·001), Bellwether capability (*β*=141·4, p = 0·049) and presence of trainees (*β*=207·5, p = 0·001). Operative volume was not correlated with availability of electricity, oxygen, light source, suction, blood, instruments, suture, gloves, intravenous fluid, or antibiotics. Composite scores for availability of infrastructure (electricity + oxygen), medications (antibiotics + IVF), and OR equipment (light + suction + instruments + suture + gloves) were created. Operative volume was not independently correlated with any of these composite scores on univariate regression. On multivariable regression analysis, operative volume was strongly predicted by number of surgical, anesthetic, and obstetric physician providers (*β*=10·72, *p* = 0·005, R^2^ = 0·94) when controlling for confounders including number of hospital beds and operating theaters, presence of trainees, and availability of infrastructure, medications, blood, and OR equipment. This regression is depicted in Fig. 4.

## Discussion

In this study, we report an annual surgical volume of 144·5 cases per 100,000 people per year in the Ugandan public sector. This finding is in line with previously published findings by Linden et al. from the year 2009–2010, suggesting surgical volume has remained stagnant in the past 7 years [13]. Operative volume in Uganda’s public sector falls far below the LCoGS recommended threshold of 5000 procedures per 100,000 population [1, 13]. At its current operative volume, Uganda’s public sector meets only 2·9% of the 2030 target set by the Lancet commission [1]. The best available evidence suggests that over 3·5 million individuals have unmet surgical need, of whom 1·4 million require surgical treatment [7].

Access to safe, timely, and affordable surgical, obstetric, and anaesthesia care is an indispensable part of health care, and yet an estimated 143 million additional surgical procedures are needed in LMICs each year to meet current surgical demand [1, 2]. Previous studies have estimated that 295 surgical procedures are performed per 100,000 persons annually in low income countries, compared to 11,110 per 100,000 in high income countries [17]. A recent surgical assessment from Madagascar reported a similar annual operative volume of 135–191 procedures per 100,000 population [9]. Operative densities are marginally higher in Liberia and Rwanda, with reported rates of 330 and 428·9 major operations per 100,000 population per year, respectively [10, 12]. In order to meet the surgical volume target and the needs of the country’s population, Uganda must employ innovative strategies to increase operative volume.

Understanding the operative case mix facilitates an understanding of the surgical procedures which are the most crucial to the health and wellbeing of a population. Obstetric and gynecological procedures, particularly cesarean section, make up the majority of surgical procedures in this series, with cesarean sections accounting for 56% of the total caseload. This is similar to the 2012 findings by Linden et al. who reported that ob/gyn cases constituted 77% of all major procedures (with cesarean sections alone accounting for 54% of the caseload), while general surgeries made up 22% of case volume, subspecialties accounting for the remainder [13]. Previous work has demonstrated that the ratio of caesarean sections to total operative volume in sub-Saharan Africa ranges from 23·3% to 41·5%, compared to a ratio of 2·6% in high income countries [22].

Notably, several LMICs have reported decreasing cesarean section rates, as surgical volumes and resources increase. In Rwanda, between 2009 and 2011, the percentage of surgical procedures that were classified as ob/gyn decreased from over 60% to 42·7% [10, 23]. Similarly, in Haiti the ratio of cesarean deliveries to total operations decreased from 13·4% to 10·7% with investment in the surgical infrastructure and corresponding increases in surgical volume [24]. Based on available evidence, Uganda has yet to undergo this transition in case mix.

The main driver of operative volume, when controlling for various confounders including hospital infrastructure, equipment, supplies, is the number of fully-trained SAO physician providers. Similar findings have been published in Sierra Leone where surgical provider density was found to be positively correlated with number of surgical procedures performed [11]. This study, however, did not assess the relationship between other factors and operative volume [11]. In order to increase operative volume in Uganda and thus increase access to safe surgical services, governments and stakeholders must increase the surgical workforce. While there are often enormous shortfalls in infrastructure and supplies at health facilities in LMICs, the most important determinant of surgical volume is the presence of a trained SAO provider [22]. This finding is unfortunate because the availability of trained surgeons, anaesthesia providers, and obstetricians is the hardest resource to produce. Training and retaining SAO providers in such resource limited contexts are challenges and these findings make a strong argument for focusing discussions on how capacity building efforts can more effectively invest in increasing the surgical workforce.

While it is not surprising that providers drive operative volume, it is important to draw attention to the finding that infrastructure, availability of equipment, oxygen, blood, and presence of trainees were not associated with operative volume in the Ugandan public sector. Available literature that details the quantitative relationship between these factors and operative volume is limited. Based on our site-specific experience, we have seen that as long as providers are present, they will attempt to improvise to compensate for deficiencies in order to provide care to desperate patients. For instance, providers often operate without oxygen or proper electricity, using head lamps to provide surgical field illumination. Moreover, work evaluating anaesthesia capacity in 22 LMICs found that over a third of facilities had no access to oxygen and no anesthetic machines yet these places continue to carry out operations [25]. It is important to note, however, that merely increasing the numbers of providers is unlikely to improve access to surgical services if not simultaneously improving the resources and infrastructure that support such complex services. Furthermore, the availability of adequate infrastructure, equipment and other inputs are likely associated with high quality and safe surgical care and thus their importance should not be underestimated as they are also crucial to providing access to safe surgical care.

It is promising to note, however, that surgical volume is rising in LMICs. In fact, comparing surgical volume in 2004 and 2012, the biggest increase in the rate of surgery occurred in LMICs [17].

### Limitations

There are several limitations to this study. First, this study scope was limited to the public healthcare sector. Analysis of the private and private not-for-profit sector has been completed and is currently being analyzed. Second, surgical volume was assessed retrospectively using data from logbooks and charts. Despite its limitations, logbooks have been found to be a simple, reproducible, and accurate method for collecting operative volume data in such a resource-limited setting [21]. Moreover, since only 30-days of the operative log were captured, this data may not reflect seasonal variation in the number of operations. Due to limitations in recording, it was not possible to identify which cases were elective and which were emergent and future studies should assess this element of case classification. Third, several of the variables were collected via interviews using the SAT, which is subject to several intrinsic methodological limitations. Fourth, we were not able to control for overlapping catchment populations or patients that may bypass facilities to seek care from a higher-level facility. Fifth, the operative volume presented here may be an underestimate given the exclusion of Mulago Hospital from the analysis. Finally, due to logistical and financial limitations, we were not able to assess all public hospitals in the country and thus may be underpowered to detect statistically significant effects within the sample.

## Conclusions

A detailed understanding of case volume and distribution is essential in facilitating targeted interventions to strengthen surgical capacity. The main driver of operative volume in Uganda is the number of fully-trained SAO physician providers. Cesarean sections continue to dominate the surgical case mix in Uganda, though it is expected that this will change with investment in infrastructure and improvements in operative volume and capacity. There is a critical need to increase the number of trained surgeons, anesthesiologists and other professionals in order to meet the surgical volume target and the needs of Uganda’s population. Human resources are undeniably one of the most critical components of surgical care provision [26]. Innovative strategies can and must be explored to facilitate this expansion of the SAO workforce, including expansion of postgraduate training and development of unique recruitment and retention strategies.

## Abbreviations

GHs: General hospitals
HMIS: Health Management Information System
IRB: Institutional review board
LCoGS: *Lancet* Commission on Global Surgery
LMICs: Low- and middle-income countries
NRHs: National referral hospitals
PGSSC: Program in Global Surgery and Social Change
RRHs: Regional referral hospitals
SAOs: Surgical, anesthetic and obstetric & gynecology physician providers
SAT: Surgical Assessment Tool
WHO: World Health Organization
